# INCLUDING COMMUNITY MEMBERS IN QUALITATIVE ANALYSIS: LESSONS FROM AN INDIGENOUS DEMENTIA PROJECT

**DOI:** 10.1093/geroni/igae098.0950

**Published:** 2024-12-31

**Authors:** Dana Ketcher, Melissa Blind, Wayne Warry, Karen Pitawanakwat, Josyaah Budreau, Melinda Dertinger, Mikaela Williams, Kristen Jacklin

**Affiliations:** University of Minnesota Medical School Duluth, Duluth, Minnesota, United States; University of Minnesota Medical School Duluth, Duluth, Minnesota, United States; University of Minnesota Medical School Duluth, Duluth, Minnesota, United States; Naandwechige-Gamig Wikwemikong Health Centre, Wikwemikong, Ontario, Canada; University of Minnesota Medical School Duluth, Duluth, Minnesota, United States; University of Minnesota Medical School Duluth, Duluth, Minnesota, United States; University of Minnesota Medical School Duluth, Duluth, Minnesota, United States; University of Minnesota Medical School, Duluth, Minnesota, United States

## Abstract

When conducting community-based participatory research (CBPR), the community can be left behind when it comes time to analyzing qualitative data. Little has been written on how qualitative research can rigorously and effectively include community perspectives and interpretations during data analysis. When conducting CBPR and/or research with historically marginalized and oppressed communities, incorporating community perspectives during analysis is especially important to assess researcher biases, de-center Western research paradigms/assumptions, and incorporate community values/worldviews. While scholars have written about incorporating Indigenous paradigms and methodologies into the various stages of research, less has been written on the exact steps teams can take to incorporate community members in the qualitative data analysis process. At our academic research center, we seek to create a more inclusive, relational, Indigenized, and decolonized approach to qualitative analysis. We have developed a qualitative analytic framework that intentionally includes community-based researchers and community advisory members, often without qualitative experience, from partner Indigenous communities. For this paper, we will describe our team’s iterative qualitative analysis approach as it relates to a multi-sited, international, ethnographic study of Alzheimer’s disease and related dementias among Indigenous North American communities in the Great Lakes region. By diversifying the perspectives present during qualitative analysis, we work to center the Six Rs of research: respect, relationship, representation, relevance, reciprocity, responsibility (Tsosie et al. 2022). As a result, we seek an ethical space to learn from both academic and community partners to ensure our approach is respectful and reflective of community and cultural values, while also being methodologically rigorous.

